# Transcriptome resequencing data for rock pigeon (*Columba livia*)

**DOI:** 10.1186/s13104-022-06007-1

**Published:** 2022-03-29

**Authors:** Hamed Kharrati-Koopaee, Ali Esmailizadeh, Fatemeh Sabahi

**Affiliations:** 1 Department of Animal Science, Faculty of Agriculture, Shahid Bahonar University of Kerman, PB 76169-133, Kerman, Iran; 2 Institute of Biotechnology, Shiraz University, Shiraz, Iran; 3 State Key Laboratory of Genetic Resources and Evolution, Kunming Institute of Zoology, Chinese Academy of Sciences, No. 32 Jiaochang Donglu, Kunming, 650223 Yunnan China; 4 Department of Plant Protection, College of Agriculture, Shiraz University, Shiraz, Iran

**Keywords:** Rock pigeon, Navigation, RNA-seq

## Abstract

**Objective:**

How do birds navigate their way? It is one of the interesting question about homing pigeons, however the genetic of navigation has reminded as a puzzle. Optic lobe, olfactory bulb, hippocampus and cere were collected for RNA sampling. The generated RNA-seq represent RNA resequencing data for racing homer (homing) pigeon and other rock pigeon breeds. The obtained data set can provide new insight about hippocampus role and GSR contribution to pigeon magnetoreception.

**Data description:**

To investigate the navigation ability of rock pigeon breeds, 60 whole transcriptome sequence data sets related to homing pigeon, Shiraz tumblers, feral pigeons and Persian high flyers were obtained. RNA extraction was performed from three brain regions (optic lobe, olfactory bulb, hippocampus) and cere. Paired-end 150 bp short reads (Library size 350 bp) were sequenced by Illumina Hiseq 2000. In this way, about 342.1 Gbp and 130.3 Gb data were provided. The whole transcriptome data sets have been deposited at the NCBI SRA database (PRJNA532674). The submitted data set may play critical role to describe the mechanism of navigation ability of rock pigeon breeds.

## Objective

Iran is a large country that contains a special range of altitudes and climates. Therefore, there is a remarkable genetic diversity of animal species [1, 2]. In Iran, due to several reasons such as decoration, food source and dry droppings pigeons have been considered for many years. Ancient pigeon houses (pigeon towers) in Iran plateau are an interesting historical example of pigeon production [3]. Next generation sequencing (NGS) platforms have revolutionized the DNA sequencing. One of the most popular platforms of NGS is whole transcriptome sequencing (RNA-seq). Whole transcriptome analysis plays a critical role in order to describe the genomic features, identifying biological pathways and networks underlying environmental challenges or different biological systems. The outcomes of RNA-seq data analysis provides new insights about biological puzzles [4]. Previously, we carried out a RNA-seq data analysis among different breeds of rock pigeons in order to explain the navigation ability of different breeds of rock pigeon in Iran [5]. Three brain regions (hippocampus, olfactory bulb, optic lobe) and also cere were selected from Persian high flyers, feral, Shiraz tumblers and homing pigeons. It should be noted that, the navigation ability of studied rock pigeon’s breeds can be classified in different groups. Persian high flyer and Shiraz tumblers are similar in navigation ability. Homing pigeon is known as racing pigeon for orientation of long distances. In contrast, feral pigeons have the weakest navigation ability. Hippocampus through complex structure contribute to learning and memory [5]. The neural basis for olfactory navigation in pigeons has been reported and also the olfactory bulb and hippocampus contribute to navigation in pigeons and mammals [6]. It should be noted that, optic lobe as a part of the midbrain, processes visual, auditory and somatosensory information. Therefore, optic lobe may critical role in navigation, however, no accurate evidence is available for this claim [7]. Various hypotheses are available about navigation and migration ability of birds such as processing spatial cues (hippocampus) [8], magnetoreception [9], olfaction (olfactory bulb), audition (auditory cortex) and visual cues (entopallium) [7]. In order to identify the differentially expressed genes in selected brain regions, 60 whole transcriptomes were sequenced from rock pigeon breeds. The provided data sets showed that the most number of differentially expressed genes (DEGs, 234) were in hippocampus. In contrast to hippocampus, few DEGs were detected in the olfactory bulb (15 genes) and the optic lobe (68 genes) between homing pigeons and other breeds [5]. The most DEGs were reported in hippocampus, for instance candidate genes such as *MFSD2A*, *KIRREL3*, *KCNAB2*, and *MAPK8IP2* were significantly up-regulated at least 1.3-fold in the homing pigeon. The outcomes of GO enrichment analysis showed that the DEGs in the hippocampus were overrepresented in terms such as cognition, learning or memory and associative learning. In addition, the potential role of GSR in pigeon magnetoreception was reported [5]. The presented data set can provide new perspective about navigation ability of rock pigeon breeds and also explanation of navigation mechanism by RNA-seq analysis.

## Data description

It should be noted that, the outcomes of transcriptome data analysis and additional methods details have previously been published in Molecular Biology and Evolution journal [5]. In order to investigate the navigation ability of rock pigeon breeds, four rock pigeon breeds, including 4 homing pigeons [30–45], 3 Shiraz tumblers [62–73], 4 feral pigeons [14–29], 4 Persian high flyers [46–61] were collected for RNA sampling from hippocampus (HC), olfactory bulb (OB) and optic lobe (OP) and cere (NS) tissues (Table 1). Pigeon samples were collected from different regions (Tehran, Marvdasht, Shiraz and Kerman) in Iran. In current study, 15 samples of rock pigeons were collected, however RNA sampling was carried out for OP, OB, NS and HC tissues (four different tissues) for each breed. In this way, the BioProject contains 60 RNA-seq samples [11]. Table 1 shows the all direct links for each tissue of rock pigeons. RNA was extracted by QIAGEN kit protocol. Agarose gel (1%) electrophoresis and Nanodrop [ratio 260/280 (nm)] were used to evaluation of the extracted RNA. Paired-end 150 bp short reads (Library size 350 bp) were sequenced by Illumina Hiseq 2000 [5]. Around 342.1 Gbp and 130.3 Gb data were provided [5] (Table 1). Btrim (version: 2.0) was applied to adaptors trimming and quality control of RNA-seq samples. Tophat2 (v2.0.13) [10] under default parameters was utilized to map the short reads to the Cliv_1.0 rock pigeon reference genome [12]. Cufflinks (v2.1.1) [13] was used to assemble the transcripts, with the cuffquant and cuffnorm programs in cufflinks used to quantify and normalize the transcript/gene expression abundances, and cuffdiff (located in the cufflinks software) used to detect differentially expressed genes using a Poisson dispersion model with a false discovery rate (FDR < 0.05). The provided data sets provide new insight about importance of the hippocampus for homing ability, and the potential role of GSR in pigeon magnetoreception.

**Table 1:** The complete RNA-seq data sets of rock pigeons at NCBI SRA database

Label	Name of data file		File type (file extension)	Data repository and identifier
Data set 1	RNA-seq of optic lobe from feral rock pigeon		Fastq (fq.gz)	NCBI SRA database https://identifiers.org/ncbi/insdc.sra:SRR10102900 [14]
Data set 2	RNA-seq of olfactory bulb from feral rock pigeon		Fastq (fq.gz)	NCBI SRA database https://identifiers.org/ncbi/insdc.sra:SRR10102916 [15]
Data set 3	RNA-seq of cere from feral rock pigeon		Fastq (fq.gz)	NCBI SRA database https://identifiers.org/ncbi/insdc.sra:SRR10102933 [16]
Data set 4	RNA-seq of hippocampus from feral rock pigeon		Fastq (fq.gz)	NCBI SRA database https://identifiers.org/ncbi/insdc.sra:SRR10102949 [17]
Data set 5	RNA-seq of optic lobe from feral rock pigeon		Fastq (fq.gz)	NCBI SRA database https://identifiers.org/ncbi/insdc.sra:SRR10102901 [18]
Data set 6	RNA-seq of optic lobe from feral rock pigeon		Fastq (fq.gz)	NCBI SRA database https://identifiers.org/ncbi/insdc.sra:SRR10102902 [19]
Data set 7	RNA-seq of optic lobe from feral rock pigeon		Fastq (fq.gz)	NCBI SRA database https://identifiers.org/ncbi/insdc.sra:SRR10102903 [20]
Data set 8	RNA-seq of olfactory bulb from feral rock pigeon		Fastq (fq.gz)	NCBI SRA database https://identifiers.org/ncbi/insdc.sra:SRR10102917 [21]
Data set 9	RNA-seq of olfactory bulb from feral rock pigeon		Fastq (fq.gz)	NCBI SRA database https://identifiers.org/ncbi/insdc.sra:SRR10102918 [22]
Data set 10	RNA-seq of olfactory bulb from feral rock pigeon		Fastq (fq.gz)	NCBI SRA database https://identifiers.org/ncbi/insdc.sra:SRR10102919 [23]
Data set 11	RNA-seq of cere from feral rock pigeon		Fastq (fq.gz)	NCBI SRA database https://identifiers.org/ncbi/insdc.sra:SRR10102936 [24]
Data set 12	RNA-seq of hippocampus from feral rock pigeon		Fastq (fq.gz)	NCBI SRA database https://identifiers.org/ncbi/insdc.sra:SRR10102950 [25]
Data set 13	RNA-seq of hippocampus from feral rock pigeon		Fastq (fq.gz)	NCBI SRA database https://identifiers.org/ncbi/insdc.sra:SRR10102951 [26]
Data set 14	RNA-seq of hippocampus from feral rock pigeon		Fastq (fq.gz)	NCBI SRA database https://identifiers.org/ncbi/insdc.sra:SRR10102952 [27]
Data set 15	RNA-seq of cere from feral rock pigeon		Fastq (fq.gz)	NCBI SRA database https://identifiers.org/ncbi/insdc.sra:SRR10102934 [28]
Data set 16	RNA-seq of cere from feral rock pigeon		Fastq (fq.gz)	NCBI SRA database https://identifiers.org/ncbi/insdc.sra:SRR10102935 [29]
Data set 17	RNA-seq of optic lobe from homing pigeon		Fastq (fq.gz)	NCBI SRA database https://identifiers.org/ncbi/insdc.sra:SRR10102907 [30]
Data set 18	RNA-seq of olfactory bulb from homing pigeon		Fastq (fq.gz)	NCBI SRA database https://identifiers.org/ncbi/insdc.sra:SRR10102924 [31]
Data set 19	RNA-seq of cere from homing pigeon		Fastq (fq.gz)	NCBI SRA database https://identifiers.org/ncbi/insdc.sra:SRR10102940 [32]
Data set 20	RNA-seq of hippocampus from homing pigeon		Fastq (fq.gz)	NCBI SRA database https://identifiers.org/ncbi/insdc.sra:SRR10102921 [33]
Data set 21	RNA-seq of hippocampus from homing pigeon		Fastq (fq.gz)	NCBI SRA database https://identifiers.org/ncbi/insdc.sra:SRR10102898 [34]
Data set 22	RNA-seq of hippocampus from homing pigeon		Fastq (fq.gz)	NCBI SRA database https://identifiers.org/ncbi/insdc.sra:SRR10102899 [35]
Data set 23	RNA-seq of optic lobe from homing pigeon		Fastq (fq.gz)	NCBI SRA database https://identifiers.org/ncbi/insdc.sra:SRR10102908 [36]
Data set 24	RNA-seq of optic lobe from homing pigeon		Fastq (fq.gz)	NCBI SRA database https://identifiers.org/ncbi/insdc.sra:SRR10102909 [37]
Data set 25	RNA-seq of hippocampus from homing pigeon		Fastq (fq.gz)	NCBI SRA database https://identifiers.org/ncbi/insdc.sra:SRR10102910 [38]
Data set 26	RNA-seq of optic lobe from homing pigeon		Fastq (fq.gz)	NCBI SRA database https://identifiers.org/ncbi/insdc.sra:SRR10102911 [39]
Data set 27	RNA-seq of olfactory bulb from homing pigeon		Fastq (fq.gz)	NCBI SRA database https://identifiers.org/ncbi/insdc.sra:SRR10102925 [40]
Data set 28	RNA-seq of olfactory bulb from homing pigeon		Fastq (fq.gz)	NCBI SRA database https://identifiers.org/ncbi/insdc.sra:SRR10102926 [41]
Data set 29	RNA-seq of olfactory bulb from homing pigeon		Fastq (fq.gz)	NCBI SRA database https://identifiers.org/ncbi/insdc.sra:SRR10102927 [42]
Data set 30	RNA-seq of cere from homing pigeon		Fastq (fq.gz)	NCBI SRA database https://identifiers.org/ncbi/insdc.sra:SRR10102941 [43]
Data set 31	RNA-seq of cere from homing pigeon		Fastq (fq.gz)	NCBI SRA database https://identifiers.org/ncbi/insdc.sra:SRR10102942 [44]
Data set 32	RNA-seq of cere from homing pigeon		Fastq (fq.gz)	NCBI SRA database https://identifiers.org/ncbi/insdc.sra:SRR10102944 [45]
Data set 33	RNA-seq of optic lobe from Persian high flyer		Fastq (fq.gz)	NCBI SRA database https://identifiers.org/ncbi/insdc.sra:SRR10102912 [46]
Data set 34	RNA-seq of olfactory bulb from Persian high flyer		Fastq (fq.gz)	NCBI SRA database https://identifiers.org/ncbi/insdc.sra:SRR10102928 [47]
Data set 35	RNA-seq of cere from Persian high flyer		Fastq (fq.gz)	NCBI SRA database https://identifiers.org/ncbi/insdc.sra:SRR10102945 [48]
Data set 36	RNA-seq of hippocampus from Persian high flyer		Fastq (fq.gz)	NCBI SRA database https://identifiers.org/ncbi/insdc.sra:SRR10102932 [49]
Data set 37	RNA-seq of hippocampus from Persian high flyer		Fastq (fq.gz)	NCBI SRA database https://identifiers.org/ncbi/insdc.sra:SRR10102943 [50]
Data set 38	RNA-seq of cere from Persian high flyer		Fastq (fq.gz)	NCBI SRA database https://identifiers.org/ncbi/insdc.sra:SRR10102946 [51]
Data set 39	RNA-seq of cere from Persian high flyer		Fastq (fq.gz)	NCBI SRA database https://identifiers.org/ncbi/insdc.sra:SRR10102947 [52]
Data set 40	RNA-seq of cere from Persian high flyer		Fastq (fq.gz)	NCBI SRA database https://identifiers.org/ncbi/insdc.sra:SRR10102948 [53]
Data set 41	RNA-seq of hippocampus from Persian high flyer		Fastq (fq.gz)	NCBI SRA database https://identifiers.org/ncbi/insdc.sra:SRR10102954 [54]
Data set 42	RNA-seq of hippocampus from Persian high flyer		Fastq (fq.gz)	NCBI SRA database https://identifiers.org/ncbi/insdc.sra:SRR10102955 [55]
Data set 43	RNA-seq of olfactory bulb from Persian high flyer		Fastq (fq.gz)	NCBI SRA database https://identifiers.org/ncbi/insdc.sra:SRR10102929 [56]
Data set 44	RNA-seq of olfactory bulb from Persian high flyer		Fastq (fq.gz)	NCBI SRA database https://identifiers.org/ncbi/insdc.sra:SRR10102930 [57]
Data set 45	RNA-seq of olfactory bulb from Persian high flyer		Fastq (fq.gz)	NCBI SRA database https://identifiers.org/ncbi/insdc.sra:SRR10102931 [58]
Data set 46	RNA-seq of optic lobe from Persian high flyer		Fastq (fq.gz)	NCBI SRA database https://identifiers.org/ncbi/insdc.sra:SRR10102913 [59]
Data set 47	RNA-seq of optic lobe from Persian high flyer		Fastq (fq.gz)	NCBI SRA database https://identifiers.org/ncbi/insdc.sra:SRR10102914 [60]
Data set 48	RNA-seq of optic lobe from Persian high flyer		Fastq (fq.gz)	NCBI SRA database https://identifiers.org/ncbi/insdc.sra:SRR10102915 [61]
Data set 49	RNA-seq of optic lobe from Shiraz tumbler		Fastq (fq.gz)	NCBI SRA database https://identifiers.org/ncbi/insdc.sra:SRR10102904 [62]
Data set 50	RNA-seq of olfactory bulb from Shiraz tumbler		Fastq (fq.gz)	NCBI SRA database https://identifiers.org/ncbi/insdc.sra:SRR10102920 [63]
Data set 51	RNA-seq of cere from Shiraz tumbler		Fastq (fq.gz)	NCBI SRA database https://identifiers.org/ncbi/insdc.sra:SRR10102937 [64]
Data set 52	RNA-seq of hippocampus from Shiraz tumbler		Fastq (fq.gz)	NCBI SRA database https://identifiers.org/ncbi/insdc.sra:SRR10102953 [65]
Data set 53	RNA-seq of hippocampus from Shiraz tumbler		Fastq (fq.gz)	NCBI SRA database https://identifiers.org/ncbi/insdc.sra:SRR10102896 [66]
Data set 54	RNA-seq of hippocampus from Shiraz tumbler		Fastq (fq.gz)	NCBI SRA database https://identifiers.org/ncbi/insdc.sra:SRR10102897 [67]
Data set 55	RNA-seq of optic lobe from Shiraz tumbler		Fastq (fq.gz)	NCBI SRA database https://identifiers.org/ncbi/insdc.sra:SRR10102905 [68]
Data set 56	RNA-seq of optic lobe from Shiraz tumbler		Fastq (fq.gz)	NCBI SRA database https://identifiers.org/ncbi/insdc.sra:SRR10102906 [69]
Data set 57	RNA-seq of olfactory bulb from Shiraz tumbler		Fastq (fq.gz)	NCBI SRA database https://identifiers.org/ncbi/insdc.sra:SRR10102922 [70]
Data set 58	RNA-seq of olfactory bulb from Shiraz tumbler		Fastq (fq.gz)	NCBI SRA database https://identifiers.org/ncbi/insdc.sra:SRR10102923 [71]
Data set 59	RNA-seq of cere from Shiraz tumbler		Fastq (fq.gz)	NCBI SRA database https://identifiers.org/ncbi/insdc.sra:SRR10102938 [72]
Data set 60	RNA-seq of cere from Shiraz tumbler		Fastq (fq.gz)	NCBI SRA database https://identifiers.org/ncbi/insdc.sra:SRR10102939 [73]

## Limitations

In the current study, only four breeds of rock pigeon were investigated and also four tissues were sampled for RNA-seq analysis. In this way, the data set could not present a comprehensive information of brain regions for navigation ability in rock pigeons.

## Data Availability

The whole genome sequence data described herein have been deposited in NCBI database as the sequence read archive (SRA) format (https://identifiers.org/ncbi/bioproject:PRJNA532674) under the accession number of PRJNA532674. Please see Table 1 and the references [14–73] for details and links to the data.

## Abbreviations

Gb: Giga byte
Gbp: Giga base pair
GO: Gene ontology
NCBI: The National Center for Biotechnology Information
DNA: Deoxyribonucleic acid
RNA: Ribonucleic acid
GSR: Glutathione-disulfide reductase
MFSD2A: Major facilitator superfamily domain containing 2A
KIRREL3: Kirre like nephrin family adhesion molecule 3
KCNAB2: Potassium voltage-gated channel subfamily A regulatory beta subunit 2
MAPK8IP2: Mitogen-activated protein kinase 8 interacting protein 2
NGS: Next generation sequencing
RNA-seq: RNA sequencing
DEGs: Differentially expressed genes
OB: Olfactory bulb
OP: Optic lobe
NS: Cere
HC: Hippocampus
